# Solvent Free Twin Screw Processed Silybin Nanophytophospholipid: In Silico, In Vitro and In Vivo Insights

**DOI:** 10.3390/pharmaceutics14122729

**Published:** 2022-12-06

**Authors:** Gasper Fernandes, Sai Lalitha Alekhya Pusuluri, Ajinkya Nitin Nikam, Sumit Birangal, Gautham G. Shenoy, Srinivas Mutalik

**Affiliations:** 1 Department of Pharmaceutics, Manipal College of Pharmaceutical Sciences, Manipal Academy of Higher Education, Manipal 576104, Karnataka, India; 2 Department of Pharmaceutical Chemistry, Manipal College of Pharmaceutical Sciences, Manipal Academy of Higher Education, Manipal 576104, Karnataka, India; 3 Scires Technologies Private Limited, Manipal-Government of Karnataka Bioincubator, Advanced Research Centre, Manipal Academy of Higher Education, Manipal 576104, Karnataka, India

**Keywords:** nanophytophospholipid, phospholipid complex, silybin, twin screw processor, in silico molecular docking and molecular dynamic studies, oral bioavailability enhancement

## Abstract

Silybin (SIL) is a polyphenolic phytoconstituent that is commonly used to treat liver disorders. It is difficult to fabricate an orally delivered SIL product due to its low oral bioavailability (0.95%). Therefore, the current research focusses on the development of a novel composition of a phospholipid complex, termed as nanophytophospholipid, of SIL by employing a unique, solvent-free Twin Screw Process (TSP), with the goal of augmenting the solubility and bioavailability of SIL. The optimised SIL-nanophytophospholipid (H6-SNP) was subjected to physicochemical interactions by spectrometry, thermal, X-ray and electron microscopy. The mechanism of drug and phospholipid interaction was confirmed by molecular docking and dynamics studies. Saturation solubility, in vitro dissolution, ex vivo permeation and preclinical pharmacokinetic studies were also conducted. H6-SNP showed good complexation efficiency, with a high practical yield (80%). The low particle size (334.7 ± 3.0 nm) and positively charged zeta potential (30.21 ± 0.3 mV) indicated the immediate dispersive nature of H6-SNP into nanometric dimensions, with good physical stability. Further high solubility and high drug release from the H6-SNP was also observed. The superiority of the H6-SNP was demonstrated in the ex vivo and preclinical pharmacokinetic studies, displaying enhanced apparent permeability (2.45-fold) and enhanced bioavailability (1.28-fold). Overall, these findings indicate that not only can phospholipid complexes be formed using solvent-free TSP, but also that nanophytophospholipids can be formed by using a specific quantity of lipid, drug, surfactant, superdisintegrant and diluent. This amalgamation of technology and unique composition can improve the oral bioavailability of poorly soluble and permeable phytoconstituents or drugs.

## 1. Introduction

The liver is the body’s primary glandular organ, controlling a diverse range of physiological and chemical process and serving as the primary organ of metabolism and detoxification [1]. Liver cells have the capacity to regenerate and recover quickly from acute and sporadic diseases [2]. However, under pathological cases such as non-alcoholic fatty liver disease, hepatitis and other liver disorders, the hepatocytes lose their regenerative potential, thus resulting in scarring, apoptosis and cirrhosis [2,3]. Long-term use of non-steroidal anti-inflammatory, cardiovascular, anticancer, antitubercular and antibiotic drugs have been attributed to hepatotoxicity [4]. Therefore, herbal-based drugs or hepatoprotective drugs which can stimulate liver cell regeneration, improve liver detoxification and improve liver function are often used in the treatment of liver diseases. Based on the mode of action, they can be broadly classified into antioxidant drugs (e.g., Silybin (SIL), rutin, quercetin), anti-inflammatory drugs (e.g., glycyrrhetinic acid), detoxification drugs (e.g., *N*-acetyl cysteine and glutathione) and hepatocyte membrane protectors (e.g., polyene phosphatidylcholine) [5]. These herbal-based medications for liver-associated diseases provide a cost-effective and low-toxicity alternative to the conventional regimen [6,7].

The treatment of liver toxicity specifically involves the use of polyphenolic phytoconstituents, in particular, flavonoids such as quercetin [8], SIL [9] and rutin [10], the most prevalent of which is SIL [11]. The hepatoprotective flavonoid ‘SIL’ is the primary constituent of silymarin, which is obtained from *Silybum marianum* and is administered via the oral route [12]. Despite the fact that SIL is readily recognised as a hepatoprotective agent, its efficacy is poor, due to its low aqueous solubility (12.9 µg/mL) and permeability (0.64 µg/mL) [13], which limit its intestinal permeability. Its low gastrointestinal absorption (20–50%) subsequently results in poor oral bioavailability (0.95% in rats) [14,15]. SIL behaves as a weak acid, with a pKa of 7.95 for the 7-OH and 6.63 for the 5-OH [16]. Although the oral route is not beneficial for SIL, numerous studies have been conducted to enhance its efficacy when taken this way, as oral administration is a frequently used drug administration route [17,18]. In order to circumvent the challenge of poor oral bioavailability, several strategies for developing nanocarriers have been utilised in the past, such as solid lipid nanoparticles, liposomes, polymeric micelles, liquid crystalline nanoparticles, solid dispersions and cyclodextrin complexes [19]. Previous studies have reported the enhancement in bioavailability of SIL (124-fold) by preparing phytosomal nanosuspensions using a thin film hydration in combination with high pressure homogenisation. Although the bioavailability was significantly increased, concerns with sophisticated instrumentation, low yield and poor drug loading can cause scaling up to be difficult [20]. In a study, Wang et al. prepared nanocrystals of SIL using wet media milling technique and encapsulated it within a mucoadhesive polyelectrolyte complex, followed by film coating with Eudragit S100 or Eudragit RL-100. They successfully enhanced the oral bioavailability by 3-fold and improved the drug loading to 35.41%, with a controlled release of the drug for over 8 h [21]. Another interesting study was conducted wherein a bioenhancer (tangeretin) was used to enhance the oral bioavailability of SIL. Among the 24 flavonoids screened, they identified tangeretin to be a potent inhibitor of the BCRP, MRP2 and P-gp efflux transporters. According to the AUC_0–t_ values, SIL A and SIL B bioavailability were enhanced by 3.62-fold and 2.68-fold, respectively. Hence, the researchers concluded that using a bioenhancer could further improve the bioavailability of drugs [22].

Recent studies using phospholipid complexes of biologically active drugs, also known as ‘Phytophospholipid complexes’ (PPC), have been proven to enhance the absorption of phytoconstituents [23,24,25]. PPCs have the potential to increase the therapeutic efficacy of phytoconstituents by altering their solubility and release characteristics, leading to enhanced permeation through the intestinal membranes. Among the various lipids, Soya phosphatidylcholine (SPC), a bio-functional surfactant, is the most commonly used lipidic carrier system for enhancing the dissolution and solubility characteristics of phytoconstituents or drugs while also being hepatoprotective in nature [26]. A complex is formed based on the structural profile of the drug and on the electron accepting and donating the nature of the oxygen and nitrogen atoms in phospholipids, respectively. Because the phenolic oxygen in flavonoids has a high tendency to accept electrons, hydrogen bonds with phosphatidylcholine are easily formed [20]. Several attempts have been undertaken in the past to augment the oral bioavailability of the active agent(s). These were conducted by producing the phospholipid complex using the conventional method of preparation by solvent evaporation [27], salting out, antisolvent precipitation, spray drying [28] supercritical fluid technology, freeze-drying and thin film hydration [24,29]. Organic solvents are often used in the preparation of phospholipid complex or PPC, which may pose problems in terms of stability, toxicity and ease of scale-up. As a result, a continuous manufacturing process without the use of toxic solvents would overcome all aforementioned disadvantages [13,20,21,22].

The twin screw process (TSP) is a continuous manufacturing process which operates under defined conditions, wherein the raw materials are forced through an orifice by controlling factors such as feed rate, temperature of the barrel and screw speed [30]. The premix forms a melt pool on entering the melting zone, thus intensifying the contact between the raw materials [31]. This technique has been reported to improve drug solubility by forming solid dispersions [32], cocrystals [33] and cyclodextrin complexes [34]. These reports also assert that TSP reduced residence time at elevated temperatures, and that screw speed improved stability by minimising oxidation and hydrolysis [35]. Hence, we hypothesised that TSP, which is a solvent-free, scalable and industrially feasible alternative to conventional methods, can form phospholipid complexes. However, prior research has shown that nanonisation presents a suitable pharmaceutical basis for enhancing the bioavailability and efficacy of drugs by increasing their surface area [36]. As a result, we identified a need for formulating a novel composition which could form nanophytophospholipids in a continuous batch process after being processed by TSP.

The present study specifically relates to development of readily water-dispersible nanophytophospholipid, using a solvent-free continuous TSP process with the goal of improving the solubility and bioavailability of SIL. The H6-SNP was characterised for various physicochemical properties, i.e., Fourier transform infrared spectroscopy (FTIR), Differential Scanning Calorimetry (DSC), Powder X-ray Diffraction (p-XRD), scanning and transmission electron microscopy. To understand the interaction between SIL and SPC, in silico studies were carried out. A reliable and sensitive bioanalytical High Pressure Liquid Chromatography (HPLC) method was established to quantify SIL in analytical samples and rat plasma. The H6-SNP was subjected to in vitro drug release, ex vivo intestinal permeation study and preclinical in vivo pharmacokinetic studies to assess the dissolution and absorption rate of SIL.

## 2. Materials and Methods

### 2.1. Chemicals and Reagents

SIL was purchased from TCI Chemicals Pvt. Ltd. (Hyderabad, India). SPC and sodium deoxycholate was procured from Sigma Aldrich, India. Kolliphor^®^ P 188 was supplied as gift sample by BASF (Mumbai, India). Sodium starch glycolate and microcrystalline cellulose (PH 101) were purchased from SD Fine Chem Ltd. (Mumbai, India). All of the other materials used were of analytical grade and were obtained from reliable sources.

### 2.2. Fabrication of SIL-Nanophytophospholipid Using Twin Screw Processor

**Figure 1:**
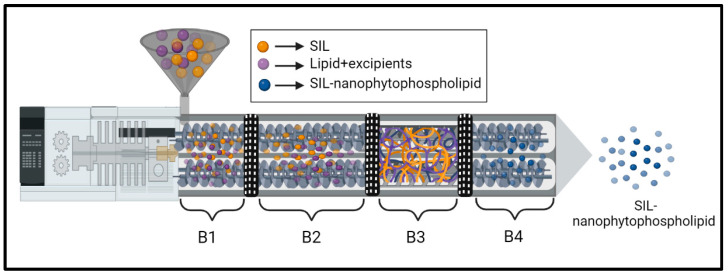
An illustration of TSP to obtain SIL-nanophytophospholipid. B1, B2, B3 and B4 represent the barrel heating zone.

The nanophytophospholipid was fabricated using a TSP (Omicron 10P, Steer Engineering, Bangalore, India) with a screw outer diameter to inner diameter ratio of 1.71. To optimise the nanophytophospholipid, the One-Factor-at-a-Time (OFAT) approach was used for development of SIL–SPC. Based on previous reports [28], SIL and SPC, at a 1:2 weight ratio, were selected and utilised to optimise the TSP parameters. The raw materials were precisely weighed (a total of 10 g), blended for 5 min and fed using a screw feeder. The screw speed of the barrel was adjusted to 200 rpm, while the temperature of the barrel heating zones were set at B1: 30 °C, B2: 100 °C, B3: 140 °C and B4: 100 °C based on the glass transition temperature of the physical mixture. An overview of the production of nanophytophospholipid, using a one-step TSP procedure, is shown in Figure 1 to facilitate understanding of this concept. The temperature in zone B1 was, by default, set to 30 °C to allow the solid feed to be conveyed to zone B2. The zone B4 was regulated to 100 °C in order to ensure that the exit product was solid in nature rather than a liquefied melt. Upon optimizing the TSP parameters to achieve SIL–SPC, the ratio of druglipid, and percentage of surfactants, superdisintegrant, bile salts and diluents were optimised with the goal of achieving the nanophytophospholipid compositions (SciSomes^TM^) shown in Table 1. The H6 batch was considered as the optimised batch (H6-SNP).

### 2.3. Dynamic Light Scattering (DLS) and Zeta Potential Analysis

After adequate re-dispersion in water, the particle size and zeta potential of the produced batches of nanophytophospholipid were assessed with DLS using the Zetasizer (Malvern Instruments, Malvern, UK) [37]. The mean of at least three experiments was used to express each particle, as well as surface charge particle information.

### 2.4. Estimation of SIL Using HPLC

The quantification of SIL in the analytical samples and rat plasma was accomplished using the HPLC method, as described earlier, with some modifications [38]. The HPLC system (Shimadzu, Kyoto, Japan) was equipped with dual piston pumps, a UV-visible detector and an auto sampler. LC solution software was used to analyse the chromatograms. The chromatographic separation was accomplished with a reverse phase C18 column (Phenomenex Luna C18). The mobile phase was made up of 15 mM KH_2_PO_4_ buffer pH 3.8 and ACN in a ratio of 54:46 *v/v*, respectively, at a flow rate of 0.8 mL/min. The analysis was carried out at 288 nm, with a total run time of 15 min. The calibration curve for the analytical samples showed good linearity, in the range of 100 to 20,000 ng/mL, with an r^2^ value of 0.999 and straight-line equation (*y* = 54,066*x* − 3414.4; where ‘*y*’ is the peak area and ‘*x*’ is the concentration of SIL). The SIL and internal standard (Naringeni (NGN)) were completely resolved in the plasma sample without any interference. The calibration curve for plasma samples was plotted in the conc. range of 25 to 15,000 ng/mL, with an r^2^ value of 0.998, indicating it was linear. The linearity equation was *y* = 0.0006*x* − 0.0079, where ‘*x*’ is the conc. of SIL and ‘*y*’ is the peak area ratio of SIL to NGN.

### 2.5. Complexation Efficiency (F%)

The amount of SIL in the produced batches of nanophytophospholipid was estimated by dispersing 10 mg of the nanophytophospholipid in methanol. The solution was sonicated for 5 min, followed by centrifugation for 10 min at 10,000× *g* rpm. After centrifugation, the supernatant was collected and filtered through a 0.22 μm filter [20,34]. This solution was diluted and analysed using HPLC method described in Section 2.4 [38]. The SIL content was determined using the Equation (1):

(1) $$F\%=\frac{Amount\;of\;SIL\;in\;the\;nanophytophospholipid}{Total\;amount\;of\;SIL\;added}$$

### 2.6. Fourier Transform Infrared Spectroscopy

The KBr pellet technique was used to analyse the IR spectra by subjecting to FTIR analysis, using Shimadzu FTIR −8300 (Kyoto, Japan). In an agate mortar, SIL, SPC, physical mixture (PM), and H6-SNP were dried and ground with KBr respectively. The dry materials were then compressed into pellets under 5 tons of pressure for 5 min, and scanned at 500 to 4000 cm^−1^ with 25 scans at a resolution of 4 cm^−1^. The FTIR graphs were collected and analysed using LabSolutions IR software [33].

### 2.7. Differential Scanning Calorimetry

The DSC for SIL, SPC, PM and SNP was performed using DSC (Shimadzu-TA-60 WS, Kyoto, Japan) to compare the peak-transition and peak onset temperatures of the samples. The samples were sealed in an empty aluminium pan, and then heated up from 25 °C to 350 °C at a speed of 10 °C per min under continuous nitrogen flow. The empty aluminium pan was used as the reference for the analysis. Heat flow was measured as a function of temperature for each sample [33].

### 2.8. Powder X-ray Diffraction

The PXRD patterns of SIL, SPC, PM and H6-SNP were obtained using an X-ray diffractometer (Rigaku Co., Tokyo, Japan). The study was carried out at ambient temperature, with *2θ* range from 5° to 80°, using a 0.02° per min scanning rate. A typical scintillation counter recorded the X-ray-diffracted beam. The current and voltage were set at 15 mA and 40 kV, respectively [33].

### 2.9. Morphological Studies

#### 2.9.1. Transmission Electron Microscopy (TEM)

The surface structure and shape of the H6-SNP were determined using TEM (FEI Tecnai G^2^ Spirit Bio-Twin, Eindhoven, The Netherlands). A drop of the diluted dispersion of H6-SNP was spread onto a carbon-coated copper grid and dried using an infrared lamp for 10 min. The dried sample was directly observed under the TEM and imaged using OSIS Veleta CCD Camera [39].

#### 2.9.2. Scanning Electron Microscopy (SEM)

The surface morphology of plain SIL and H6-SNP was investigated using a scanning electron microscope (EVO MA18 Oxford EDS (Zeiss, Oberkochen, Germany). A 10 torr vacuum was applied after the samples were deposited on the aluminium stub, which was made conductive by gold sputtering. This was followed by scanning the samples with an electron beam, while the SEM photomicrograph images were collected using the SmartSEM software [40].

#### 2.9.3. Energy Dispersive Spectroscopy (EDS)

The elemental mapping or purity of a substance can be determined using Energy Dispersive Spectroscopy (EDS). SEM-EDS (EVO MA18 Oxford EDS (Zeiss, Germany)), equipped with an EDS detector, was used to determine the chemical elements present in H6-SNP. A acceleration voltage of 20 kV was applied to the samples after fixing on carbon double-faced adhesive tape [40].

### 2.10. Molecular Docking and Molecular Dynamic (MD) Studies of SIL–SPC Complex

In order to elucidate the binding interaction of SIL with SPC, the Maestro module (version 10.7) of the Schrodinger software suite (Schrodinger, LLC, New York, NY, USA) was utilised to examine the molecular docking and dynamics.

#### 2.10.1. Molecular Docking Studies

The LigPrep module (version 5.5, Schrödinger), equipped with Epik (version 5.5, Schrödinger) [41], was used to optimise the structure of SIL (obtained from PubChem CID: 31553) [42], which was processed at pH 7.4 to obtain the ionized state of the molecule. The Macro model (version 13.1, Schrödinger), equipped with an OPLS4 force field [43], was utilised to optimise the geometry of the SPC molecule, which was fetched from Protein data bank (PDB ID: 3B7Q) [44]. Before the optimization, SPC was refined in order to add the missing hydrogen, and the water molecule was removed using the protein preparation wizard [45]. The processed structures of SIL and SPC were molecularly docked using Maestro’s GLIDE module (grid-based ligand docking with energetics) in XP mode to obtain the glide score, using Equation (2) [46].

(2) Glide Score=Ecoul+EvdW+Ebind+Epenalty

The binding affinity “ΔG” was computed using the Prime MM-GBSA module. Several properties, such as electrostatic π–π packing, lipophilicity, Van der Waals, strain and columbic energies, were calculated for the complex and individual components. As stated in Equation (3), the total ΔG was determined using energy-minimised parameters [47].

(3) $$\Delta G=E_{SIL-SPC\left(minimized\right)}-E_{SIL\left(minimized\right)}-E_{SPC\left(minimized\right)}$$

#### 2.10.2. MD Simulations and Stability Analysis

The MD simulation was conducted using the Desmond module (6.6) to investigate complex formation, with the complex being placed inside an orthorhombic simulation box with a solvent thickness layer of 10 Å, using the TIP4P explicit water module. The MD simulations were carried out for 100 ns, at 1.013 bar and 300 K using the Matrtyna–Tobias–Klein barostat and the Nose–Hoover chain thermostat, respectively. The trajectory generated was recorded at an interval of 20 picoseconds (ps), and after simulation, RMSD plots were generated to analyse the stability of the SIL–SPC complex [48,49].

### 2.11. Equilibrium Solubility Studies

According to the previously described technique [33,34], equilibrium solubility experiments on SIL, PM and H6-SNP were implemented using several buffer solutions of differing pH values, i.e., pH 1.2 (HCl buffer), pH 4.5 (sodium acetate buffer), pH 6.8 and 7.4 (phosphate buffer), pH 9.2 (alkaline borate buffer) and water. A surplus amount of plain SIL and H6-SNP was added into a 2 mL microcentrifuge tube containing different buffer solutions and water. The tubes were spun at 50 rpm for 48 h using a tube rotator (Maas Instruments, Mumbai, India). The solution was centrifuged at 15,000× *g* rpm for 15 min, then, the supernatant was collected and filtered through a 0.22 μm filter and analysed using HPLC [38] to determine the content of SIL using the method described in Section 2.4.

### 2.12. Partition Coefficient Studies

The partition coefficient study was carried out using the Yue et al. approach, with some alterations [50]. In 10 mL of water, SIL, PM and H6-SNP (equivalent to 200 mg of SIL) were added respectively and agitated for 24 h. The solution was collected in a separating funnel after centrifugation for 10 min at 10,000× *g* rpm. To this solution, n-octanol (10 mL) was added and agitated for 12 h. Both phases were separated, filtered using a 0.22 μm filter and analysed using HPLC [38] in order to quantify the concentration of SIL in both phases, using the method described in Section 2.4. The log P was computed, utilizing Equation (4).

(4) $$\mathrm{Log}\;P=\log\;[\frac{Co}{Cw}]$$

C_o_ = conc. of SIL in n-octanol phase

C_w_ = conc. of SIL in the aqueous phase

### 2.13. In Vitro Drug Release Study

The in vitro drug release test for SIL, PM and H6-SNP was conducted according to the type II USP dissolution test method [34]. The dissolution media were HCl buffer (pH 1.2; with 0.5% Tween 80) and phosphate buffer (pH 6.8; with 0.5% Tween 80), intended to replicate the pH of the stomach and intestinal fluid. The 1000 mL dissolution flask was filled with 900 mL of dissolution media and immersed in a water bath. The paddle speed was set at 75 rpm. At the start of the study, SIL, PM and H6-SNP (≈35 mg of SIL) were added to the dissolution media. The samples were taken out from the flask at 0.25, 0.5, 0.75, 1 and 2 h for pH 1.2 buffer, and at 0.5, 1, 2, 4, 6, 8 and 10 h for pH 6.8 buffer, then subsequently replenished with fresh buffer solution (10 mL), respectively. The samples were filtered and analysed to determine the content of SIL using the HPLC method, as described in Section 2.4 [38].

### 2.14. Ex Vivo Studies Using Everted Rat Ileum Sac Method

Male Wistar rats weighing 200–250 g were used in the experiments. The animals were housed at Manipal’s Central Animal Research Facility. The IAEC, Kasturba Medical College, Manipal authorised the experimental (protocol IAEC/KMC/95/2021). Animal handling was carried out in compliance with institutional and national norms for animal care and use of animals. The study was conducted using the everted rat ileum sac model. The ileum portion of the rat was removed and flushed with pH 7.4 Ringer’s solution. A glass rod was used to evert the ileum, with one end knotted and the other knotted with thread, after injecting 1 mL of Ringer’s solution. The assembly was incubated in 50 mL of Ringer’s solution, and was termed as the mucosal compartment. The perfusion apparatus was termed as the serosal compartment, wherein the SIL and H6-SNP suspension (≈ 5 mg/mL of SIL) was added in the beaker. The experimental solution was continuously aerated and maintained at 37 °C. The sample was withdrawn at regular intervals (10, 20, 30, 40, 50, 60, 90, 120 and 180 min), up to 3 h [51]. The extracted volume was replenished by fresh Ringer’s solution, and the concentration of SIL in the serosal compartment was analysed using the HPLC method described in Section 2.4 [38].

### 2.15. In-Vivo PK Studies

Male Wistar rats were subjected to fasting overnight before administering the drug, and were then divided into two groups: Group I: Plain SIL (200 mg/kg; p.o.); Group II: H6-SNP (200 mg/kg; p.o.). The drug or H6-SNP (≈200 mg/kg of SIL) was dispersed in water and administered orally. Prior to blood withdrawal, rats were anesthetised in an isoflurane-filled chamber. At time intervals of 0.5, 1, 2, 4, 8, 12 and 24 h, 200 µL of blood was drawn from each rat’s retro-orbital plexus and placed in a centrifuge tube containing 10% EDTA. After 10 min of centrifugation at 10,000× *g* rpm, 100 μL of the plasma was separated and stored at −20 °C [20,34].

#### 2.15.1. Extraction of SIL from Plasma

The plasma samples were processed utilizing a modified liquid–liquid extraction method (LLE) to extract SIL, as previously described by Chi et al. [20]. Into an aliquot of 100 μL of thawed plasma, 5 μL of NGN (internal standard) was incorporated and vortexed, followed by addition of chilled ACN and tert-butyl-methyl-ether (TBME) in a ratio of (1:40 *v*/*v*). The solution was vortexed, followed by centrifugation at 10,000× *g* rpm for 10 min. After separating and evaporating the organic phase at 40 °C, under continuous nitrogen flow, the residue was reconstituted with 100 μL of the mobile phase. The samples were eluted using the HPLC conditions mentioned in Section 2.4.

#### 2.15.2. Data Processing and Statistical Treatment

The maximum plasma concentration (C_max_), area under the plasma concentration time curve (AUC), elimination half-life (K_el_), absorption half-life (t_1/2_) and mean residence time (MRT) were calculated using PK Solutions software (PK Solutions 2.0^TM^). The data were statistically analysed by one-way ANOVA followed by Tukey’s post hoc tests, using Graph Pad Prism version 8.0.1 (San Diego, California, USA) A *p* value of less than 0.05 was considered statistically significant.

## 3. Results

### 3.1. Formulation of Nanophytophospholipid

Phospholipids are a key component of the cell membrane, and are low in toxicity and biocompatible. Because phospholipids act as absorption enhancers or solubilisers, they are frequently employed to improve drug permeability [52]. Numerous studies have shown that complexation with phospholipids enhances the oral bioavailability of several poorly soluble phytoconstituents, such as SIL [53], quercetin [54], curcumin [55], ellagic acid [56], rutin [57], marsupsin [58], oxymatrine [50], naringenin [59], chrysophenol [60], embelin [61], catechin [62] and puerarin [63]. Researchers, likewise, discovered that the biological activity was significantly improved. However, the poor dispersive nature of the phospholipids in water can be overcome by formulating a readily dispersible nanosuspension using TSP, thereby overcoming the conventional solvent-based method of preparation. It should be noted that phospholipids act as a molten carrier, which serves as an alternative to conventional solvents, wherein the molten matrix acts as a viscous solvent accommodating the parent molecule. In our study, we used a solvent-free TSP to obtain nanophytophospholipids. In this process, two parameters, namely barrel temperature and screw speed, are vital to the development of drug lipid complexes. These variables can be altered to enhance the interaction between the molten carrier and the drug. However, unlike the solvents which evaporate, in TSP, the molten polymer forms a matrix as it exits the extruder. Hence, it highly favours the chance for amorphization of the drug.

The OFAT method was employed for optimization. It involved altering one process parameter at a time while holding the others constant. A 1:2 molar ratio of SIL:SPC was selected based on previous reports [28] in order to optimise the TSP parameters based on the outcome, i.e., solubility and appearance of the complexes. The formation of the SIL–SPC complex is a prerequisite for the formation of nanophytophospholipids.

Initially, we varied the temperature of the barrel heating zones. We observed that a specific set of barrel temperature in four heating zones of the TSP was necessary to form complexes (B1: 30 °C, B2: 100 ± 5 °C, B3: 140 ± 3 °C, B4: 100 ± 5 °C). If the temperature exceeded the set temperature of the B3 zone, granules with discoloration were formed.

The impact of screw speed was examined after the temperature was set, and it was found that at lower rpm (100), large sized granules were formed. However, by increasing the rpm to 200, the residence time was reduced, further resulting in fine granular complexes. The feed rate had minimal influence on the appearance of the complexes.

Once the parameters of TSP were set, the drug-to-phospholipid ratio was varied, with the goal of achieving low particle size, higher complexation efficiency, and good solubility. In order to form nanophytophospholipids of SIL, it was necessary to include excipients, which were necessary to form a stable dispersion in nanometric dimensions. The complexation efficiency reduced with the increase in SIL concentration, hence increasing the phospholipid concentration and leading to higher complexation of SIL, as shown in Table 2. Thus, a 1:3 weight ratio of SIL:SPC was selected for further optimization due to the higher complexation. Even though phospholipid is a natural stabiliser, it is inadequate to disperse in water; therefore, a surfactant, i.e., P188 was added at 0.5% to enhance the physical stability and reduce the particle size. The formulation of H1, H2 and H3 had formed sticky granules with poor dispersibility in water, as shown in the polydispersity index data in Table 3. Hence, a super-disintegrant (i.e., SSG) was added to cause an immediate dispersion in water, forming a stable nanosuspension. The formation of a dispersible nanosuspension was accredited to the concentration of surfactant (nanometric dimension) and superdisintegrant (immediate dispersion). In order to overcome the sticky nature of the granules, an adsorbent, i.e., MCC, was added, which improved the practical yield, as shown in Table 2. The practical yield was considerably increased from 59.2% (without MCC; H4) to 80% (with MCC; H6). Several reports [64,65,66] claim that the addition of bile salt improves the stability of vesicular delivery vehicles in the GIT by producing micelles, thus increasing the solubility and avoiding precipitation. Hence, SD was added in the final optimised nanophytophospholipid (H6-SNP) [52].

### 3.2. Particle Size, PDI and Zeta Potential

The formulated nanophytophospholipid batches of SIL were dispersed in water, and then subjected to particle size and zeta potential analysis using the Malvern Zetasizer. The results of size, zeta potential and PDI are shown in Table 3. When SPC and P188 were present at a higher concentration, the particle size was the lowest, i.e., the SNP (H6) showed a particle size of 334.7 ± 3.0 nm with a PDI value of 0.18, implying that the particles were uniform in nature. Earlier studies have shown that particles greater than 5 µm are up-taken by the lymphatic system, and particles below 500 nm can transverse the cell membranes via endocytosis [67]. When determining particle stability in a suspension, the zeta potential is a crucial factor to consider [68]. A sterically stabilised system has a zeta potential of −30 mV or higher. The optimised H6-SNP exhibited a zeta potential value of −30.21 ± 0.3 mV, indicating that the particles are highly stable and have a low tendency to aggregate. The results in Table 3 show that increasing the concentration of lipids and surfactants reduces particle size and lowers the PDI. These findings suggest that the H6-SNP was comparatively homogeneous and stable.

### 3.3. Fourier-Transformed Infrared Spectroscopy

Previous literature has shown that phospholipid complexes are neither new chemical compounds nor physical mixtures alone, but are associated with some weak interactions, such as weak Van der Waals force or hydrogen bonding. Additionally, the absence of solvents in TSP makes it impossible for it to undergo any chemical reaction to form new substances.

**Figure 2:**
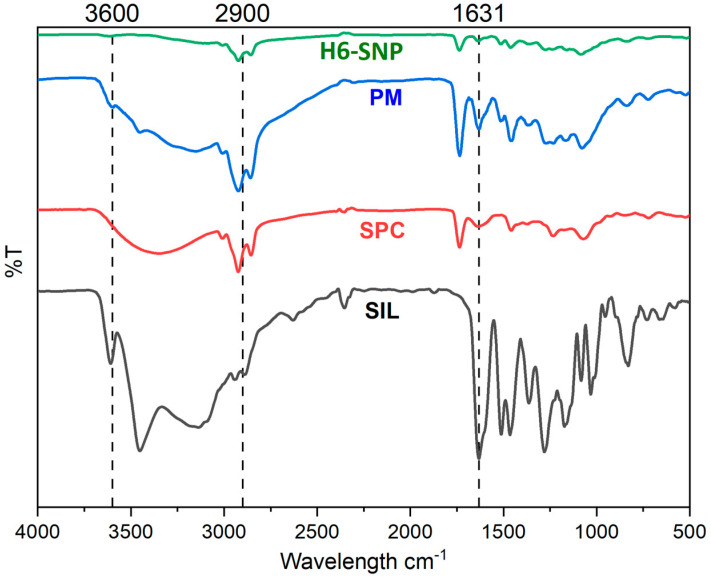
A Multiview Fourier Transform Infrared spectrum of SIL, SPC, PM and H6-SNP.

The FTIR spectrum for SIL, SPC, PM (containing SIL, SPC, poloxamer188, sodium starch glycollate, sodium deoxycholate and microcrystalline cellulose) and H6-SNP is shown in Figure 2. The potential molecular interactions between SIL and SPC at the solid state were explored here. SIL had distinct peaks for –OH and –CH, stretching between 3400 and 2900 cm^−1^, C-C stretching, and C=O stretching between 1700 and 1000 cm^−1^. The difference observed in the FTIR pattern of H6-SNP was identical to that of SPC, with no characteristic peaks of SIL between 1700 and 1000 cm^−1^, indicating that a new composite was formed. The presence of a new peak, at 2924 cm^−1^ in the FTIR spectrum of H6-SNP, verified the establishment of H-bonding between the C=O groups of SPC and free OH groups of SIL, while the disappearance of peaks at 1631 cm^−1^ (C-O stretching), due to shielding by the phospholipid molecule by intermolecular coupling, indicated the formation of a new co-amorphous state. Eventually, we observed that additional conjugation bonds were not formed between SIL and SPC, but rather, weak intermolecular interactions, such as ‘H’-bonding between the -OH of SIL and -C=O and -PO_3_^2−^ carboxyl and phosphate groups in SPC, were formed. The characteristic peaks of SPC and SIL were retained in the PM, suggesting the absence of any chemical interactions. The reduced intensity of the peak may be due to the presence of other formulation components. Our findings are consistent with earlier research, in which the phospholipid complex was generated using different phytoconstituents [54,57,69].

### 3.4. Differential Scanning Calorimetry

**Figure 3:**
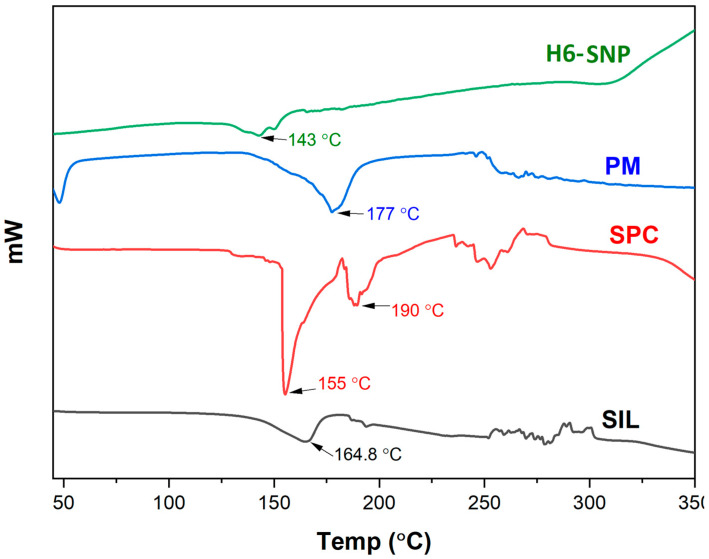
A Multiview Differential Scanning Calorimetry thermograms of SIL, SPC, PM and H6-SNP.

DSC offers maximum data regarding the physical interaction and compatibility between the drug and components of the formulation. These interactions, particularly, are notably linked to the shift in the onset temperature or melting point, the change in peak shape or enthalpy and the disappearance or appearance of endothermic peaks. The DSC thermograms for SIL, SPC, PM and H6-SNP is shown in Figure 3. The principal aim of DSC studies was to examine the thermal behaviour of SIL in its complex and plain forms. SIL exhibited a melting endotherm at 164.8 °C, signifying that the drug is crystalline and comparable to that reported in the literature [70]. The melting endotherm of SPC showed a minor peak at 190 °C, which could be attributed to the movement of the polar head in the phospholipid molecular structure, as well as a sharp peak at 155 °C, which could be attributed to SPC’s phase transition from a gel to a liquid crystalline state. In addition, the phospholipid must have gone through melting, isomeric or other crystalline changes [71]. The PM showed a broad, undefined and fused peak at 177 °C, which may be due to miscibility of SIL in the phospholipid during the slow heating process, with partial formation of the complex. The peak observed at 50 °C may be ascribed to the movement of the polar heads of the phospholipid chain [55,55,72]. According to prior research, decreasing the enthalpy and melting endotherm has been proven to reduce the drug’s crystallinity and increase drug solubility [73]. In the thermogram of the complex, a single broad peak is seen at 143 °C, which differs from the melting endotherm of SIL and SPC. The peaks of SIL and SPC disappear form the complex, and a lower phase transition temperature is seen as compared to SPC. These observations indicate the possible formation of the complex, wherein the conversion of the SIL’s crystalline to amorphous form must have taken place [74]. There are hydrophobic, Van Der Waals and hydrogen bonding interactions between the drug and SPC, which is evident from the in silico studies and has also been reported in previous literature [53,55]. The interaction of SIL with the polar part of the hydrocarbon tail of the SPC molecules causes it to rotate freely and encapsulate the drug. As a result, the SPC hydrocarbon chain sequence shortens, the peak of SPC vanishes and the phase transition temperature drops. Hence, the absence of peaks and the decrease in the phase transition temperature of the H6 conclude the formation of the complex [20].

### 3.5. Powder X-ray Diffraction

**Figure 4:**
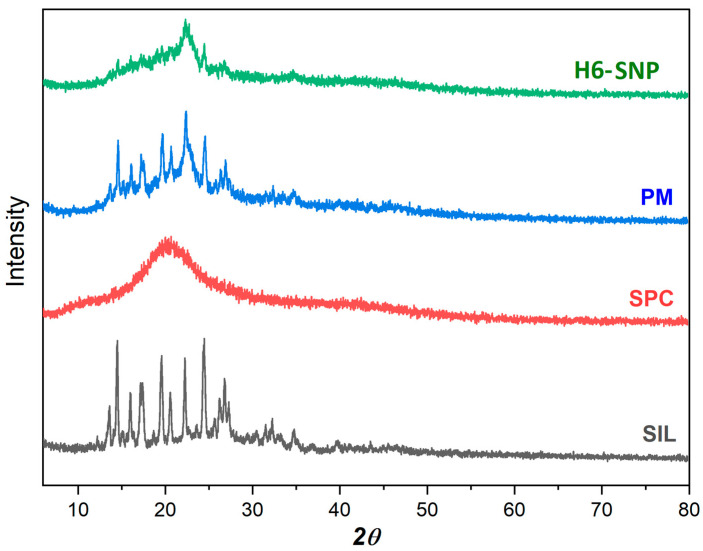
A Multiview Powder X-Ray Diffraction p-XRD diffractograms of SIL, SPC, PM and H6-SNP.

PXRD is one of the most used techniques for assessing possible changes in the internal state of a drug crystal [72]. The main objective here was to assess the solid-state form of SIL in the complex. Figure 4 depicts the PXRD spectra of SIL, SPC, PM and H6-SNP. Numerous diffraction peaks between 10° *2θ* and 30° *2θ* were seen in the X-ray spectra of SIL, demonstrating its crystalline nature. The PXRD of SPC exhibited no discernible diffraction peaks, indicating the amorphous nature of the compound. The PM showed reduced intensity of the diffraction peaks of SIL, indicating that drug was present in crystalline form and had limited interaction with the excipients. The presence of other formulation components may have had a diluting effect on SIL, causing its reduced intensity. However, the diffraction peaks of SIL disappeared in the X-ray spectrum of the complex, indicating the conversion of the crystalline form of the drug into the amorphous form. These findings are consistent with previous studies on phospholipid drug complexes [20,72,75]. This assertion that the drug amorphised in the complex is further supported by the DSC data.

### 3.6. Morphological Studies by Electron Microscopy

#### 3.6.1. Transmission Electron Microscopy (TEM)

**Figure 5:**
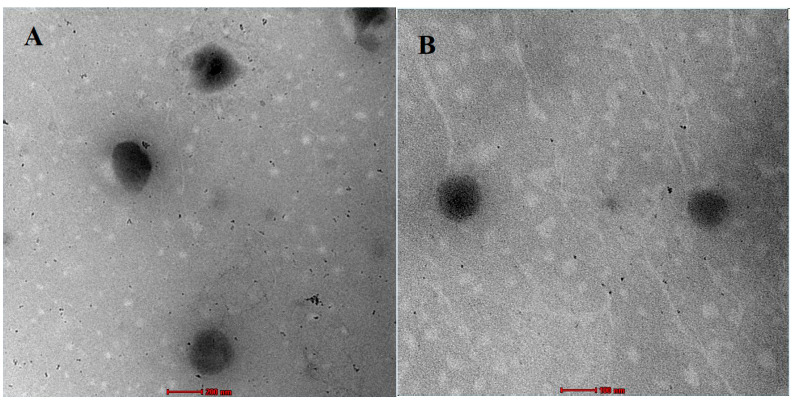
TEM images of H6-SNP. (**A**) Represents lipid nanoparticles (H6-SNP) H6-SNP at 200 nm scale. (**B**) Represents micelles, which are additionally found along with H6-SNPs, at 100 nm scale.

The surface morphology of the H6-SNP was characterised by TEM, and is shown in Figure 5. The TEM images confirmed the formation of distinct and almost spherical nanosized particles of H6-SNP. The TEM images (Figure 5A) showed a particle size of about 300–350 nm, which is in accordance with the particle size obtained with the DLS technique using the Zetasizer. Moreover, an interesting observation was made, wherein spheroidal micelles were also observed (Figure 5B) with a size of about 100 nm. These micelles might have been formed due to the presence of sodium deoxycholate in the nanophytophospholipid composition. Sodium deoxycholate, a bile salt, improves the enzymatic stability, chemical stability and permeation of the nanoparticle in the GIT. The formation of micelles has also been confirmed in previous reports when sodium deoxycholate was used [64,65,66]. However further characterisation studies are required to confirm the formation of micelles along with H6-SNP composition.

#### 3.6.2. Scanning Electron Microscopy (SEM)

**Figure 6:**
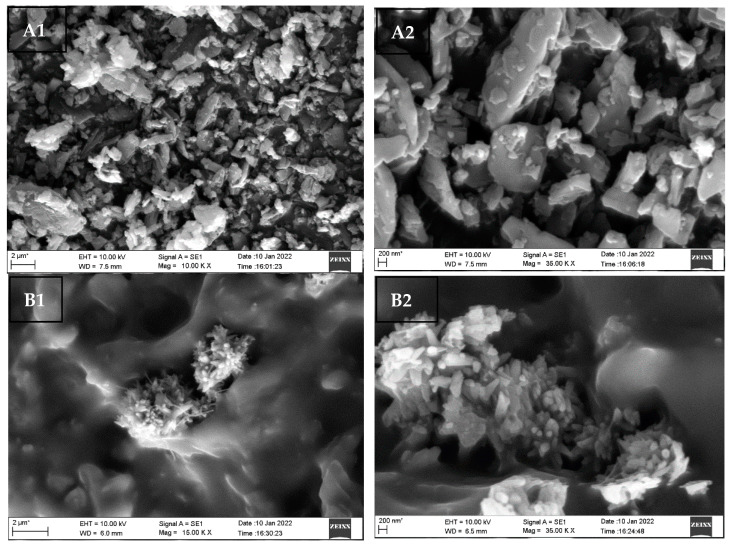
Scanning Electron Microscopy images of SIL and H6-SNP. (**A1**,**A2**) represent SIL images at 2 µm and 200 nm scale; (**B1**,**B2**) represent the H6-SNP images at 2 µm and 200 nm scale.

The SEM images of SIL and H6-SNP, at 2 µm and 200 nm, are shown in Figure 6. The surface of SIL looks to be irregular and somewhat crystalline, with a smooth surface. In contrast, the H6-SNP exhibited a major morphological change, with a rough and porous surface enveloping SIL, which may contribute to the increased dissolution rate and solubility of SIL.

#### 3.6.3. Energy Dispersive Spectroscopy (EDS)

**Figure 7:**
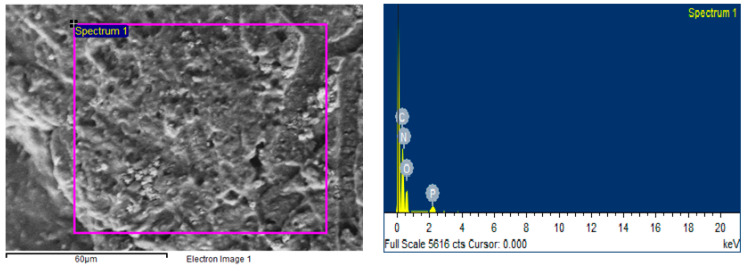
Elemental Mapping of H6-SNP using EDS.

EDS is a microanalytical technique equipped with SEM to validate the purity of a material by detecting the total composition of the chemical elements present in the samples. The results of EDS for the H6-SNP show the presence of carbon, nitrogen, oxygen and phosphorus, which are the elements present in SPC (molecular formula-C_35_H_66_NO_7_P), SIL (molecular formula C_25_H_22_O_10_) and the remaining formulation components (SSG, MCC, SD and P188). Hence, the results show an absence of any contaminants in the H6-SNP. The elemental composition is presented in Table 4 and represented in Figure 7.

### 3.7. Molecular Docking and Dynamic Simulation Studies of SIL-PPC Complex

#### 3.7.1. MD Docking and Pose Analysis

**Figure 8:**
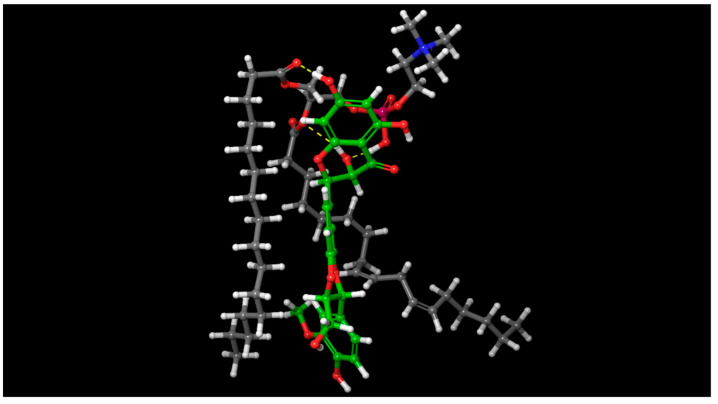
Interaction bonds and binding observed with SIL–SPC complex; Blue: Aromatic hydrogen bonds; Yellow: Hydrogen bonds; Purple: Halogen bonds.

The goal of the molecular docking investigation was to figure out how the SPC and SIL may interact on the formation of the SIL–SPC complex (prerequisite for the formation of SIL-nanophytophospholipid). Figure 8 shows the best docking position, based on the docking score of −3.09 and XP G score of −3.09.

**Figure 9:**
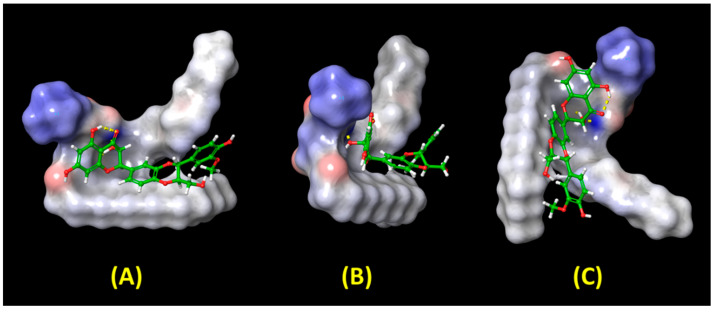
Illustration of the binding poses of SIL and SPC on forming complexes in three different orientations, (**A**–**C**).

Halogen bonds, hydrogen bonds and aromatic hydrogen bonds were found to be involved in the interaction between the SPC and the SIL. The hydrophobic part of the SIL–SPC complex is encircled by two hydrophobic arms of SPC and one hydrophilic OH of SIL, which forms H bonding with the phospholipid’s -P=O structure. The binding poses of SIL and SPC which form complexes in the three best orientations re shown in Figure 9.

Table 5 shows the data acquired upon running Prime MM-GBSA, which was used to determine the “ΔG bind” based on the strains found between SIL and SPC (based on Equation (2)). The “ΔG bind” contributions from the lipophilic interactions, Van der Waals, coulomb interaction and hydrogen bonds were found to be −43.51 kcal/mol, −22.69 kcal/mol, −15.2 kcal/mol and −0.67 kcal/mol respectively. As a result of the molecular docking pose analysis, hydrogen bonding and aromatic hydrogen bonds. owing to the benzene rings. are key contributors to the formation of the complex.

**Figure 10:**
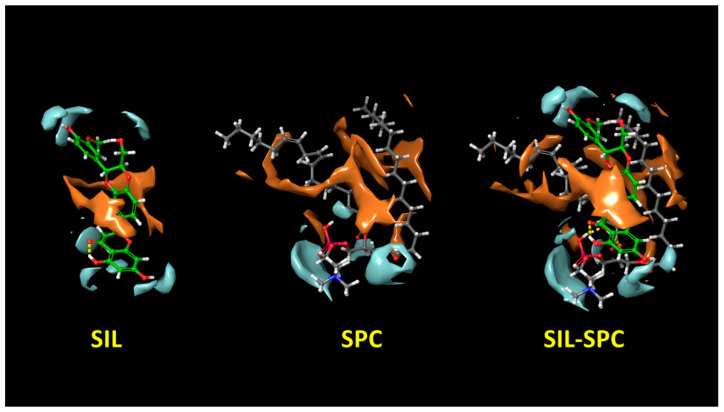
Hydrophilic (blue) and hydrophobic surface (brown) of SIL, SPC and SIL–SPC complexes.

For the purpose of promoting the permeation and dissolution of a molecule, its hydrophilic and hydrophobic surfaces should be higher. In the study, SIL and SPC had a hydrophilic surface area of 112.76 Å^2^ and 122.09 Å^2^, respectively, and a hydrophobic surface area of 99.03 Å^2^ and 256.35 Å^2^, respectively. Upon complexation, the hydrophilic and lipophilic surface area of the SIL–SPC complex increased, and was found to be 192.02 Å^2^ and 290.75 Å^2^, respectively. The hydrophilic and lipophilic surface area of the complex area rose by 1.7-fold and 2.93-fold, respectively, compared to SIL, and by 1.57-fold and 1.13-fold, respectively, when compared to SPC. Figure 10 makes it evident that the lipophilic and hydrophilic surface areas of the complex were enhanced.

#### 3.7.2. MD Simulation and Trajectory Analysis

**Figure 11:**
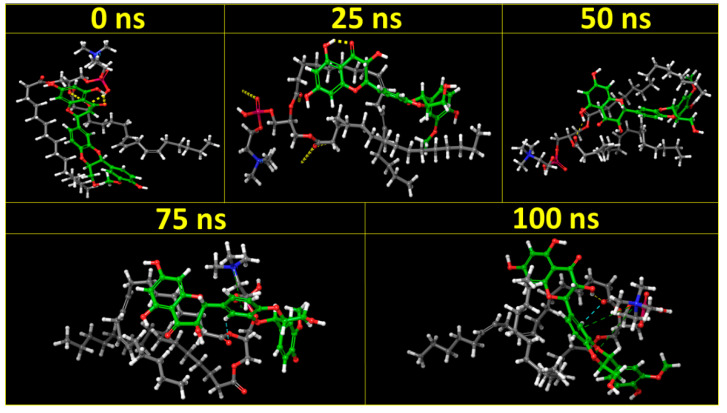
Molecular dynamic simulation of SIL–SPC complex at 0, 25, 50, 75 and 100 ns.

MD simulations were carried out for a period of 100 ns to examine the stability of SIL in the SIL–SPC complex. The end configurations and the initial structural poses at 0, 12, 25, 50 and 100 ns are shown in Figure 11. At different time intervals, images indicated different spatial configurations and unique structures of the SIL–SPC complex, implying that the starting and ending structural positions are different. The MD trajectory diagrams indicated the fabrication of a complex, with slight deviations in SIL’s beginning and ending orientations in the complex. The ‘H’ bond between SIL and SPC was retained throughout the 100 ns simulation.

**Figure 12:**
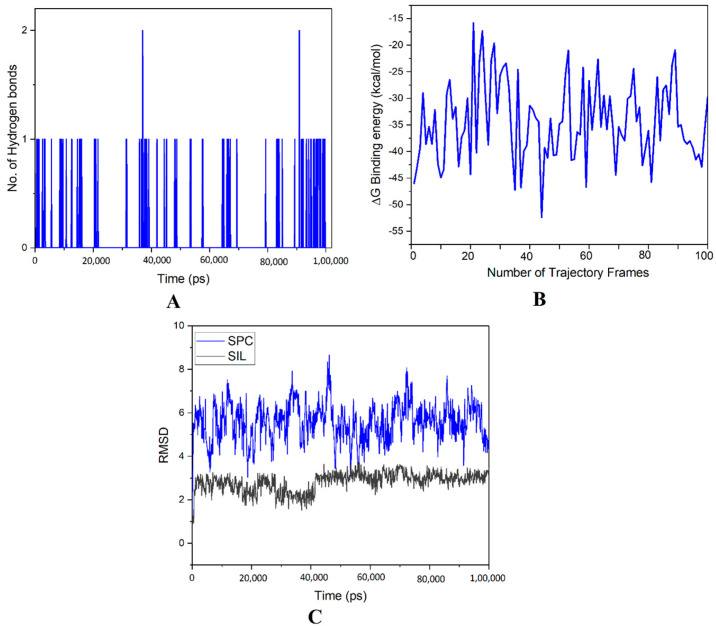
MD simulations of SIL–SPC complex. (**A**) Trajectory frames number; (**B**) hydrogen bonds number; (**C**) root mean square deviation plot.

The formation of low energy complexes was suggested by the MD simulation trajectory, which was corroborated by high binding energy. As shown in molecular docking, the free binding energies of SIL and SPC, estimated using MMG-GBSA MD simulation trajectory frames (Table 5), indicated a strong contribution of lipophilicity and Van der Waals interaction (Figure 12B).

Understanding the precise interaction between SPC and SIL is aided by the no. of H- bonds obtained from MD simulation trajectory frames. Figure 12A depicts the H-bonds’ formation between SPC and SIL, with changes in conformation at 0, 25, 50 and 100 ns. Polar interaction graphs were generated to examine the changes in hydrogen bond patterns throughout the 100 ns MD simulation. A pulsative appearance of hydrogen bonds was seen, with a nearly similar distribution in these plots, except that the H-bond varied between 1 and 2 at 37 ns and 90 ns.

The complex’s stability was examined using the first frame’s trajectory, aligned to SPC atoms, while the RMSD for SPC and SIL was computed with respect to the initial frames. The RMSD plots are shown in Figure 12C, and reflect the complex’s stability. A value within 4 Å suggests the generation of a stable complex. Therefore, in the present study, Figure 12C shows that the complex lies within the RMSD bracket of ≤4.0 Å.

### 3.8. Equilibrium Solubility Studies

The equilibrium solubility of SIL, PM and H6-SNP was assessed in water and buffers of varying pH values, as shown in Table 6. SIL is a poorly soluble, weak acid, and exhibits pH-dependent solubility with low aqueous solubility (7.72 ± 0.16 μg/mL) [16]. The PM showed better solubility in comparison to pure SIL (3.71-fold), which may be due to the presence of surfactants and lipids, which can enhance the solubility of the drug. The H6-SNP showed significantly high solubility in the entire pH range compared to pure SIL and PM, i.e., pH 1.2 (33.98-fold and 10.97-fold), pH 4.5 (32.14-fold and 9.77-fold), pH 6.8 (66.7-fold and 16.12-fold), pH 7.4 (68.25-fold and 119.8-fold), pH 9.0 (13.27-fold and 7.84-fold) and water (68.84-fold and 18.41-fold). The SIL decreased molecular crystallinity. In addition, the phospholipids’ amphiphilic nature, which can form quasi-stable bonds with the hydrophobic moiety of SIL, could explain the increase in the solubility of the drug. Additionally, hydrogen bonds involving the SIL phenolic hydroxyl group increase its affinity to water. The increase in solubility of the nanophytophospholipid is another indication of the formation of complexes by weak intermolecular interactions, and the nanonisation of the readily dispersible suspension in water and buffers of varying pH values.

### 3.9. Partition Coefficient Study

The n-octanol/water partition coefficient study for pure SIL, PM and H6-SNP was investigated. The drug had a log P of 0.73, which was comparable with the findings reported by Zeng et al. [13], whereas the PM and H6-SNP had a log P of 0.82 and 1.79, respectively (Table 7), indicating the enhanced lipophilicity of the nanophytophospholipid in comparison to the drug and PM. Before a drug permeates through the GI tract membranes, it must first dissolve in the intestinal or stomach fluid. The aqueous solubility of SIL increased by several times when formulated into nanophytophospholipids, according to equilibrium solubility experiments. The partition coefficient study also revealed that, in addition to hydrophilicity, the complex’s lipophilicity had risen, implying that the H6-SNP would immediately disperse into nanometric dimensions and would readily pass across membranes, contributing to an increase in the bioavailability. These data are in accordance with the in silico derived model, which shows the enhanced lipophilic and hydrophilic nature of the SIL–SPC complex.

### 3.10. In Vitro Drug Release Studies

**Figure 13:**
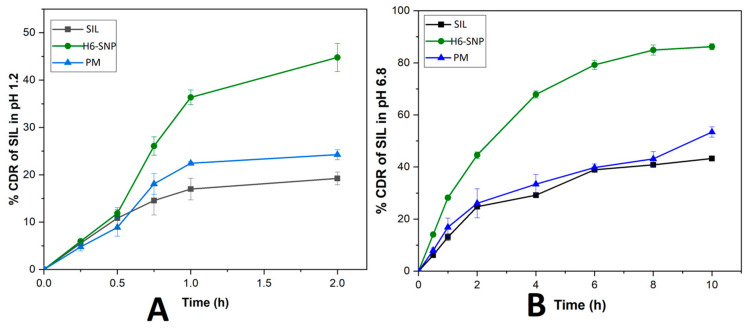
In vitro dissolution profile of SIL, PM and H6-SNP at pH 1.2 (**A**) and pH 6.8 (**B**). The results are expressed as mean ± SD, *n* = 3.

The in vitro drug release studies on SIL from plain SIL, PM and H6-SNP were carried out in pH 1.2 HCl buffer and pH 6.8 phosphate buffer in order to mimic the simulated conditions of the stomach and intestinal pH, respectively [51]. According to the release study’s findings, as shown in Figure 13A,B, the dissolution rate of SIL from H6-SNP was much higher than that of PM and plain SIL, at pH 1.2 and pH 6.8. The percentage of SIL dissolved was 19.23 ± 1.34% in pH 1.2 buffer in 2 h and 43.25 ± 0.49% in pH 6.8 in 10 h. A similar pattern of drug release was observed with PM (24.26 ± 1.06% in pH 1.2 buffer in 2 h and 53.43 ± 1.96% in pH 6.8 in 10 h) with a slight increase in the dissolution rate when compared to plain SIL. This modest increase in the dissolution rate could be caused by the formulation excipients (P188, SPC and MCC) present in the PM. However, the dissolution profile of H6-SNP showed an enhanced rate of dissolution, ascribed to the improved solubility of SIL in the nanophytophospholipid. Approximately 44.78 ± 2.97% and 86.21 ± 1.14% of the SIL was released from H6-SNP in pH 1.2 and pH 6.8 buffer, respectively. As shown in Figure 13, a biphasic release pattern was observed for H6-SNP at pH 6.8, with approximately 60% of drug released in 4 h due to an initial burst release, followed by a gradual release pattern over the next 6 h. The initial burst release may be due to extensive interaction of the water molecules with SIL and other formulation excipients, i.e., P188, SSG and MCC, causing rapid solubilisation of the drug from the nanophytophospholipid surface [72]. The sustained drug release pattern may be due to slow diffusion of SIL from the lipid bilayers of the nanophospholipid dispersion [76,77].

Prior research has shown that phospholipid molecules function as amphiphilic surfactants, improving the dispersibility and wettability of the drug [78]. Additionally, nanonisation increases the drug’s surface area, which increases the rate at which it dissolves, since it has more contact with the dissolution media. Moreover, the mechanical force generated due to the intermeshing rotating screws of the TSP ensures homogenous distribution of SIL and other excipients, which also promotes the amorphization of SIL, as evident in the DSC and PXRD studies [34]. This amorphization of SIL in the nanophytophospholipid had a positive impact on cumulative drug release. The H6-SNP dissolution rate was higher, at pH 1.2 and 6.8, indicating that the nanophytophospholipid can be orally administered to achieve rapid release of SIL in both stomach and intestinal pH conditions [20]. Hence, the novel composition of H6-SNP prepared by TSP can be considered as an efficient method to augment the dissolution rate of SIL.

### 3.11. Ex Vivo Intestinal Permeation Studies

**Figure 14:**
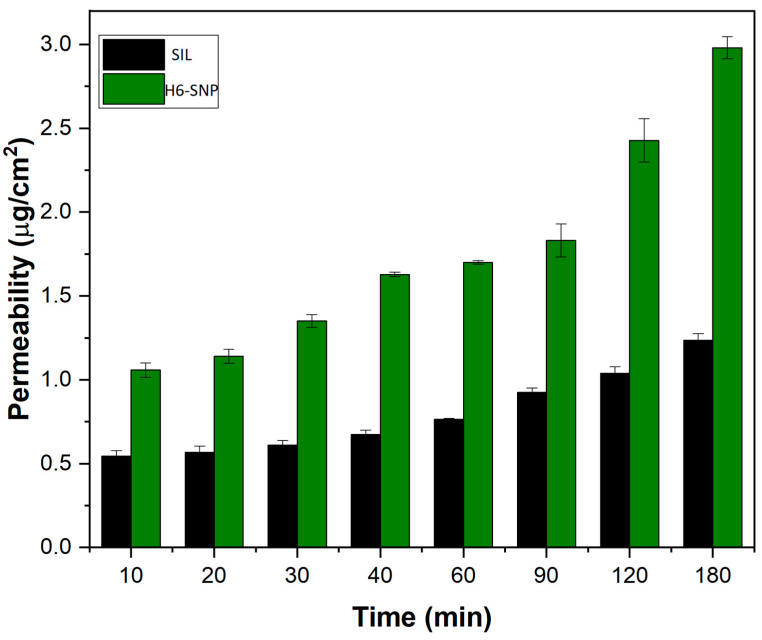
Ex vivo permeation studies of SIL and H6-SNP. The results are expressed as mean ± SD, *n* = 3.

The ex vivo everted rat intestinal model for determining drug permeability using apparent permeability values is a cost-effective, quick and reproducible approach [79]. Figure 14 shows the results of the 3 h ex vivo permeability analysis of SIL and H6-SNP. The P_app_ for SIL was 0.64 × 10^−5^ cm/min and H6-SNP was 1.57 × 10^−5^ cm/min, i.e., a 2.45-fold increase in permeation. The permeation of SIL from the H6-SNP across the rat ileum was found to be higher compared to SIL alone. The amphiphilic nature of the phospholipid, which may itself act as a surfactant, allows the drug to traverse across the membrane. The lipidic nature and nanometric dimension of nanophytophospholipids may be other reasons for enhanced intestinal membrane permeability. The increased concentration gradient of SIL across the intestinal lumen, due to its enhanced aqueous and lipid solubility in its complex form, might also have contributed to the improved intestinal permeation of SIL from H6-SNP.

### 3.12. In Vivo PK Studies

SIL exhibits limited in vitro and in vivo biological properties. Due to their limited solubility in aqueous and lipid environments, high molecular weight polyphenols have inadequate oral absorption, resulting in poor diffusion across enterocyte lipid membranes. Furthermore, SIL is quickly metabolised by Phase II biotransformation enzymes in the liver [14,70]. Because of these restrictions, it is critical to evaluate the complex’s in vivo performance, which is explained below.

**Figure 15:**
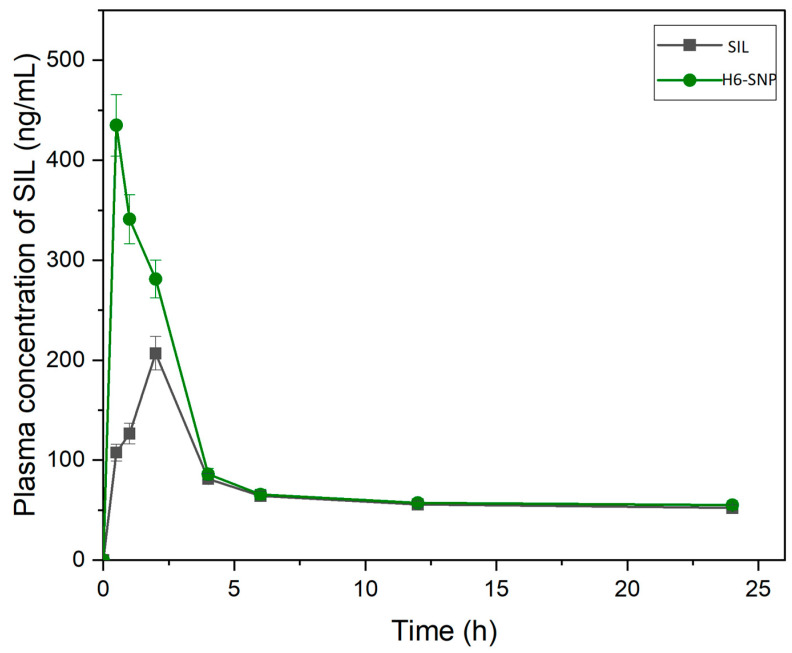
In vivo pharmacokinetic plasma concentration vs. time profile of SIL vs. H6-SNP (mean ± SD; *n* = 6).

When compared to SIL alone, the H6-SNP showed an enhanced in vivo pharmacokinetic profile. Figure 15 depicts the concentration–time graph of SIL following oral delivery of plain SIL and H6-SNP. The C_max_ and t_max_ for H6-SNP, after a single oral dosage of SIL at 200 mg/kg, were 435.1 ± 28.24 ng/mL and 0.5 h, respectively. The C_max_ observed with H6-SNP was enhanced by 2.1-fold compared with the C_max_ of plain SIL. The PK profile was also obtained by using a physical mixture as well; however, the PK profile hardly changed in comparison with plain SIL, and hence, the results are not presented. Previous investigations have also reported almost similar PK profiles for PM, as well as for the pure drug [20,80]. The difference in the C_max_ and t_max_ values between plain SIL and H6-SNP was statistically significant (*p* < 0.05). The MRT and t_1/2_ values of H6-SNP formulation were greater than those of plain SIL, indicating that SIL, in nanophytophospholipid form, has a prolonged residence time in the body. A significant increase in the AUC_0–24_ (1.28-fold) was observed with H6-SNP when compared with plain SIL, thus suggesting an improvement in oral bioavailability (PK parameters are shown in Table 8). This enhanced oral bioavailability is attributed to: (i) increased lipid solubility of SIL upon forming nanophytophospholipids, (ii) increased intestinal permeability, (iii) nanonisation, (iv) enhanced aqueous solubility of SIL and (v) increased concentration gradient across intestinal membrane, thereby improving the passive diffusion of the drug across the GI membrane. Yanyu et al. [53] used the conventional solvent evaporation technique to prepare the phospholipid complex of SIL, with C_max_ and AUC of 126.72 ng/mL and 1220.33 ng/mL × h, respectively. However, when compared to the conventionally prepared phospholipid complex of SIL, the H6-SNP we prepared using TSP had higher C_max_ and AUC, respectively.

As a result, we can deduce that TSP can be used to formulate not only phospholipid complexes, but also nanophytophospholipids, by using a particular combination of the excipients (surfactants, diluents and disintegrant) with improved dissolution profiles and pharmacokinetic characteristics. The oral bioavailability enhancement of the H6-SNP is still limited in comparison to its high solubility (66.7-fold) and improved permeation (2.45-fold), which may be due to the metabolism of SIL due to phase I and phase II enzymes as well as efflux transporters, thereby preventing the drug from being absorbed further [81]. Therefore, we can assume that including efflux transport inhibitors in the composition might also aid in augmenting the bioavailability of SIL [22]. However, the improved pharmacokinetics will improve the therapeutic efficacy of SIL, which can result in enhanced anti-inflammatory and hepatoprotective activity against various liver disorders, such as viral or drug induced hepatitis and cirrhosis, or alcohol- or drug-induced liver diseases [82]. To support this claim, further studies using an appropriate animal model should be conducted.

## 4. Conclusions

In the current study, we successfully prepared phospholipid complexes of SIL using the TSP process without the use of organic solvents. Additionally, we developed nanophytophospholipids utilizing a novel composition to address SIL’s restricted preclinical applicability, due to its poor solubility and bioavailability. The H6-SNP showed enhancement in its yield, complexation efficiency and aqueous and lipid solubility. Moreover, nanonisation led to an increase in the permeability and dissolution rate of SIL. The in vivo studies clearly depicted an enhancement in the oral bioavailability of SIL. Meanwhile, in order to understand the diffusional behaviour of the nanophytophospholipid, microparticle tracking analysis in hydrogel at pH 6.8 could be conducted in future studies to simulate the intestinal mucus. In addition, in vitro hepatoprotective activity and pharmacodynamic studies in an appropriate animal model will be required to establish the therapeutic efficiency of the generated SIL. Given the phospholipid complex’s numerous benefits, this prototype technology, along with this composition, can be used to manufacture nanophytophospholipids of potential poor soluble and permeable drugs or phytoconstituents. This technology will be useful for scale-up and large-scale manufacturing, since its manufacturing process is continuous.

## 5. Patent

The work reported in this manuscript has been filed for a patent to Patent Office Government of India, Chennai, India (Application Number 202241055257; date of filing: 27 September 2022). The nanophytophospholipid composition reported in this manuscript has been applied for a trademark as SciSomes^TM^ to The Registrar of Trade Marks, The Office of the Trade Marks Registry, Chennai, India (Application No: 5700172 & 5700173; date of filing: 28 November 2022).

## Figures and Tables

**Table 1:** Optimization of working formula to prepare nanophytophospholipid batches of SIL using TSP.

Components	Role	H1	H2	H3	H4	H5	H6	H7	H8
SIL *	Drug	1	1	1	1	1	1	1	1
SPC *	Lipid	2	1	3	3	3	3	3	3
Poloxamer 188 (P188)	Surfactant	0.5%	0.5%	0.5%	0.5%	0.5%	0.5%	0.5%	0.5%
Sodium Starch glycolate (SSG)	Super disintegrant	-	-	-	0.2%	0.2%	0.2%	0.2%	0.2%
Microcrystalline cellulose (MCC)	Adsorbent	-	-	-	-	20%	20%	-	-
Talc	Adsorbent	-	-	-	-	-	-	20%	-
Aerosil	Adsorbent	-	-	-	-	-	-	-	20%
Sodium deoxycholate (SD)	Bile salt; aids permeation	-	-	-	-	-	0.25%	-	-

* Weight ratio.

**Table 2:** Practical yield and complexation efficiency of nanophytophospholipid batches of SIL.

Batches	Practical Yield (%)	Complexation Efficiency (%)
H1	36.4	48.99 ± 1.00
H2	42.6	45.73 ± 0.57
H3	54.8	56.49 ± 0.48
H4	59.2	57.77 ± 0.51
H5	78.0	79.90 ± 0.38
H6	80.0	81.24 ± 0.80
H7	65.2	67.27 ± 1.04
H8	55.8	60.46 ± 1.01

**Table 3:** Particle size, PDI and zeta potential of nanophytophospholipid batches of SIL.

Batch	Particle Size (nm)	Zeta Potential (mV)	PDI *
H1	453.2 ± 5.0	−39.3 ± 1.52	0.44 ± 0.04
H2	490.8 ± 2.2	−37.3 ± 0.40	0.48 ± 0.02
H3	337.6 ± 4.0	−36.2 ± 1.72	0.28 ± 0.03
H4	418.0 ± 2.2	−34.9 ± 0.21	0.43 ± 0.01
H5	374.0 ± 4.3	−32.8 ± 1.13	0.37 ± 0.02
H6	334.7 ± 3.0	−30.2 ± 0.30	0.18 ± 0.00
H7	445.1 ± 3.0	−35.3 ± 0.20	0.58 ± 0.03
H8	520.0 ± 3.8	−32.6 ± 0.51	0.55 ± 0.02

Values are reported as mean ± SD, *n* = 3. * PDI = Polydispersity Index.

**Table 4:** Elemental composition of H6-SNP.

Element	Weight %	Atomic %
Carbon	59.15	65.82
Oxygen	39.51	33.00
Nitrogen	1.14	1.09
Phosphorus	0.2	0.09
Total	100%	

**Table 5:** ‘ΔG bind’ energy for the SIL–SPC complex, determined by Prime MM-GBSA calculations for molecular docking and MD simulation.

Parameters	ΔG Values in kcal/mol						
	ΔG Bind *	ΔG Bind Colomb *	ΔG Bind H Bond *	ΔG Bind Lipo *	ΔG Bind vdW *	ΔG Bind Covalent *	ΔG Bind Solvation *
Molecular docking	−65.80	−15.20	−0.67	−43.51	−22.69	6.85	9.43
MD simulation	−34.45	−8.30	−0.15	−16.01	−21.87	0.54	11.35

* ‘G bind’: binding affinity; ‘G bind Colomb’: coulombic energy; ‘G Bind H Bond’: hydrogen bond energy; ‘G Bind Lipo’: lipophilic bond energy; ‘G Bind vdW’: the Van der Waals interaction energy.

**Table 6:** Equilibrium solubility studies of SIL, PM and H6-SNP in water and different pH values.

Different Media	Concentration of Pure SIL (μg/mL)	Concentration of SIL in PM (μg/mL)	Concentration of SIL in H6-SNP (μg/mL)
0.1 N HCl buffer (pH 1.2)	2.52 ± 0.25	7.8 ± 0.36	85.64 ± 1.97
Sodium acetate buffer (pH 4.5)	4.73 ± 0.32	15.56 ± 0.4	152.06 ± 1.69
Water	7.72 ± 0.16	28.66 ± 3.05	531.46 ± 1.67
Phosphate buffer (pH 6.8)	9.30 ± 1.27	38.5 ± 1.11	620.72 ± 1.84
Phosphate buffer (pH 7.4)	12.83 ± 1.18	44.22 ± 4.26	875.66 ± 1.03
Alkaline borate buffer (pH 9.0)	80.13 ± 1.6	135.5 ± 2.78	1063.63 ± 1.34

All values are reported as mean ± SD; *n* = 3.

**Table 7:** Partition coefficient of SIL, PM and H6-SNP.

Parameters	SIL	PM	H6-SNP
C_o_ (µg/mL)	636.26 ± 9.45	1128.54 ± 6.64	5596.02 ± 45.09
C_w_ (µg/mL)	117.19 ± 2.29	166.95 ± 0.94	88.97 ± 2.60
P (C_o_/C_w_)	5.42	6.75	62.89
Log P	0.73	0.82	1.79

**Table 8:** Pharmacokinetic profile of SIL and H6-SNP.

PK Parameters	Plain SIL	H6-SNP
C_max_ (ng/mL)	206.9 ± 12.01	431.1 ± 28.24 *
T_max_ (h)	2.00 ± 0.00	0.50 ± 0.00 *
AUC_0–24h_ (h∗ng/mL)	1693.52 ± 90.16	2175.69 ± 148.57 *
AUC_0–∞_ (h∗ng/mL)	6652.04 ± 146.88	8373.88 ± 354.46 *
K_el_ (h^−1^)	0.04 ± 0.00	0.03 ± 0.00 *
t_1/2_ (h)	65.73 ± 1.12	78.01 ± 2.38 *
MRT (h)	91.06 ± 1.26	103.17 ± 2.79 *

All values are expressed as mean ± SD; *n* = 6. AUC, area under the curve; t_1/2_, elimination half-life; K_el_, elimination rate constant, MRT, mean residential time. * *p* value less than 0.05 (statistically significant) in comparison with plain SIL.

## Data Availability

Not applicable. The data may be available on request.
